# Accurate and instant frequency estimation from noisy sinusoidal waves by deep learning

**DOI:** 10.1186/s40580-019-0197-y

**Published:** 2019-08-15

**Authors:** Iman Sajedian, Junsuk Rho

**Affiliations:** 1 Department of Mechanical Engineering, Pohang University of Science and Technology (POSTECH), Pohang, 37673 Republic of Korea; 2 Department of Materials Science and Engineering, Korea University, Seoul, 02842 Republic of Korea; 3 Department of Chemical Engineering, Pohang University of Science and Technology (POSTECH), Pohang, 37673 Republic of Korea

**Keywords:** Frequency estimation, Deep learning, Neural networks

## Abstract

We used a deep learning network to find the frequency of a noisy sinusoidal wave. A three-layer neural network was designed to extract the frequency of sinusoidal waves that had been combined with white noise at a signal-to-noise ratio of 25 dB. One hundred thousand waves were prepared for training and testing the model. We designed a neural network that could achieve a mean squared error of 4 × 10^−5^ for normalized frequencies. This model was written for the range 1 kHz ≤ f ≤ 10 kHz but also shown how to easily be generalized to other ranges. The algorithm is easy to rewrite and the final results are highly accurate. The trained model can find frequency of any previously-unseen noisy wave in less than a second.

## Introduction

Estimation of the frequency *f* of a noisy sinusoidal wave has been one of the main problems in the field of signal processing and communications, due to its vast applications including power systems [1], communications [2], and radar [3–5]. Many theoretical techniques have been proposed to solve this problem; examples include discrete Fourier transform [6–9], least square**s** methods [10–12] and phase-locked loops [13, 14]. All of the proposed methods are focused on speed and accuracy of the estimation.

Recently deep learning has helped humans in many different areas of science, including medical diagnoses [15, 16], speech recognition [17, 18], photonics [19–22] and image classification [23, 24].

Deep learning which already had been introduced in the field of signal processing [25] has also helped researchers in many areas such as for finding the ideal ratio mask estimation for filtering out the noise from a spectrogram [26], real time frequency monitoring [27], channel detection in orthogonal frequency-division multiplexing systems [28, 29], and in getting channel state information feedback from massive multiple–input multiple-output systems [30] as a few examples.

Here, we show how a deep-learning algorithm can find *f* of a sinusoidal wave that is polluted by Gaussian noise. Neural networks (NNs), which belong to the family of deep-learning methods, can derive meaningful results from complicated and complex problems, and may detect patterns that human beings do not see in data; finding *f* of a noisy signal is a good example of such a problem. We know that a noisy signal is related to its *f*, but mathematical identification of that relation can be difficult. NNs can find this *f* with reasonable accuracy and high speed. Once an NN model has been trained, it can find *f* of any new given wave in less than a second. So our proposed method can easily replace the traditional analytical methods that are currently used by a neural network model, with the advantages of having higher accuracy and a faster estimation.

## Methods

We start by defining the problem. We want to find *f* of a noisy sinusoidal wave

1 $$S(t) = A\sin (2\pi ft + \varphi ) + \varOmega (t),$$

where *A* is amplitude, *t* is time, $\varphi$ is phase, and *Ω* is zero-mean Gaussian noise with a variance of σ^2^. So the *S(t)* which is the noisy wave will be our input and the *f* which is the frequency will be our output. The signal-to-noise ratio (SNR), which shows the quality of the signal, is the ratio of signal power *P*_S_ to noise power *P*_N_ [9]:

2 $$SNR = \frac{{P_{S} }}{{P_{N} }} = \left( {\frac{A}{\sigma }} \right)^{2} ,$$

and can be expressed in decibels as

3 $$SNR_{dB} = 10\log_{10} \left( {SNR} \right) = 10\log_{10} \left[ {\left( {\frac{A}{\sigma }} \right)^{2} } \right],$$

which gives us the variance as

4 $$\sigma^{2} = \frac{{A^{2} }}{{10^{{\frac{{SNR_{dB} }}{10}}} }}.$$

So given *SNR*_*dB*_ and *A* we can obtain the variance that is needed for calculating the noise function.

### Neural network

Now we discuss the neural network architecture that we used to solve this problem. We explain the process in two parts. First, we discuss the details of data preparation and data preprocessing needed for the model to work more efficiently and also the validation process that assures that the model works for the unseen data. Then we discuss the model design that we used.

#### Data preparation

In NNs, we need three datasets to assure that the model works for any new unseen data. These datasets are named training, validation, and testing dataset. The training dataset is used to train the model at each step. The validation dataset is the first unseen data; this set is used to check the model at each step, specifically to tune the hyperparameters of the model to get the lowest possible loss in predicted results. Once the best model is found (the one that has the lowest loss on the validation dataset), it is checked one more time on the test dataset to assure that the model works on any unseen data. This step assures that the model was not biased to work for the validation dataset, and so works for any new unseen data [31]. We prepared 100,000 waves for the whole dataset; we used 72% of the waves as the training dataset, 18% as the validation dataset, and 10% as the test dataset.

We considered the range 1 kHz ≤ *f* ≤ 10 kHz. For each wave, we took 2000 samples from each generated wave in each *f* in 1-μs time steps from 0 to 2000 μs; i.e., our whole dataset was a 100,000 × 2000 array. This means that the input layer of our neural network should have 2000 nodes. Since we want to find the frequency of each wave, the output layer of our neural network should only have 1 node, which corresponds to the frequency sought.

Neural networks work better if their output is between 0 and 1 or in other words if their output is normalized, so we divided the output layer by the maximum *f* = 10 kHz before the training starts. This made our new output range from $0. 1 { }\left( { = \frac{{1 {\text{KHz}}}}{{10 {\text{KHz}}}}} \right)$ to $1\left( { = \frac{{10 {\text{KHz}}}}{{10 {\text{KHz}}}}} \right)$. We multiplied all results by 10,000 after the training is finished to recover the correct values.

#### Network design

**Fig. 1:**
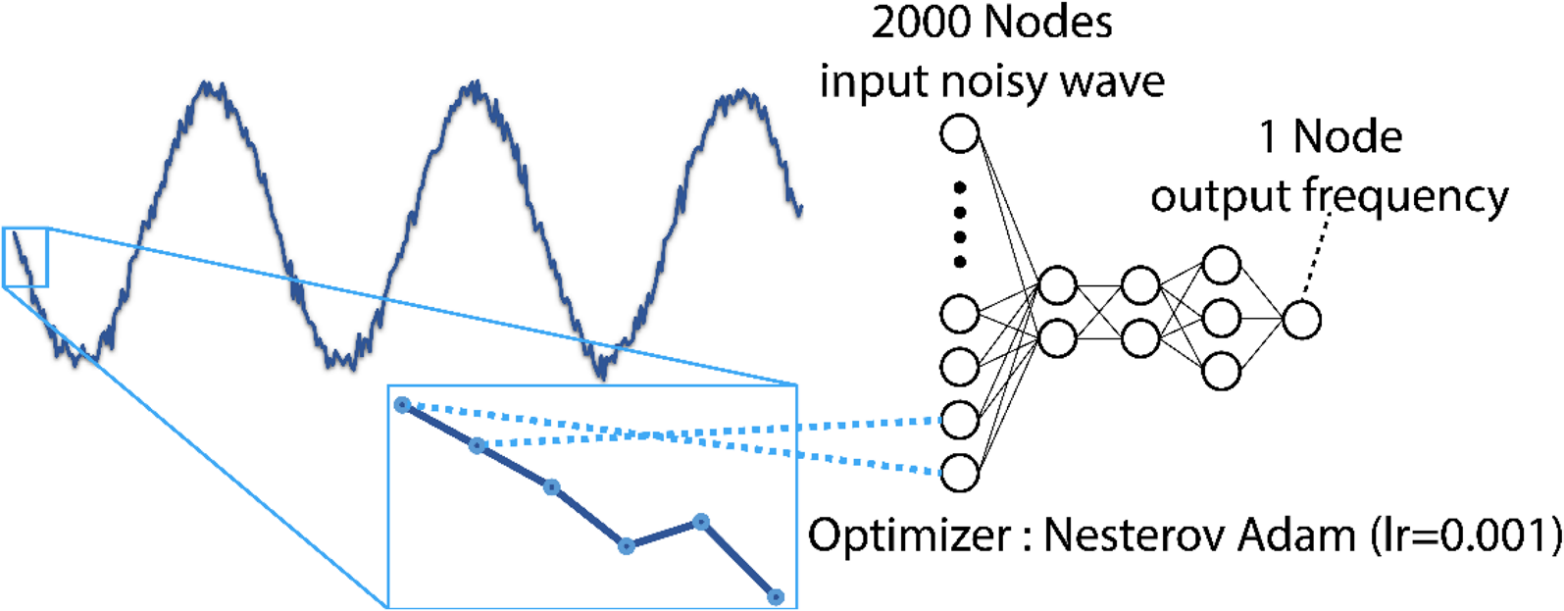
Schematic of the neural network (NN) model. To prepare the noisy sine wave as the input of the NN model we took 2000 samples from each wave. Each data sample is a node in the input layer of the NN model as is shown here. The NN model has three hidden layers with 2, 2, and 3 neurons in the first, second and third, respectively. The output layer has only one node, which represents the desired frequency. The Nesterov Adam optimizer with a learning rate (lr) of 0.001 was used for this model

We used a three-layer network with 2, 2, 3 neurons in the first, second, and third hidden layer respectively (Fig. 1 right). This architecture was found after trying many designs; this one had the lowest loss on the validation dataset. We had to use a very small number of neurons to prevent the model from overfitting. Many methods can be used to prevent overfitting; examples include using dropout (or other types of regularization), or reducing the complexity of the model, or increasing the amount of data [32]. We found that reducing the complexity of the model had the best effect and led to very good results. For other hyperparameters of the network, we used the Nesterov–Adam optimizer with a learning rate of 0.001; the metric to measure the loss of the model was mean squared error

5 $$MSE = \frac{1}{n}\sum\limits_{i = 1}^{n} {\left( {Y_{i} - P_{i} } \right)}^{2} ,$$

where *n* is the number of measurements, *Y*_*i*_ are the real values and *P*_*i*_ are the predicted values. All codes were written in Python with the help of TensorFlow and Keras packages. Calculations were performed on a computer with a 4-core 3.50-GHz processor, 32 GB of RAM, and an NVIDIA GTX 750Ti GPU with 2 GB GDDR5 RAM. The procedure of preparing the data and training the final model took less than 2 h on this computer. The trained model can predict new results in less than a second.

## Results

**Fig. 2:**
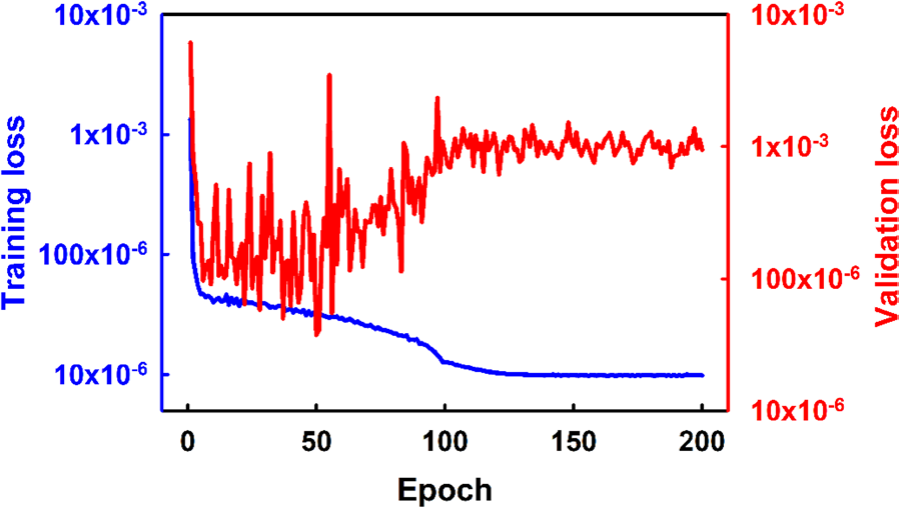
Training loss and validation loss as the model trains. The code monitors the model’s progress and saves the model with the lowest loss on the validation dataset as the best model. Both of the y-axis curves are shown on a logarithmic scale

We set *A* = 1 and $\varphi$ = 0 (Eq. 1). We also set $SNR_{dB} = 25$ as a typical setting for a good signal, which leads to $\sigma = 0.5$ [4]. The lowest recorded losses were 9.71818 × 10^−6^ on the training dataset (at epoch190) and 3.75632 × 10^−5^ on the validation dataset was (at epoch 50) in normalized values. The evaluated loss on the test dataset was 1.87709 × 10^−4^ in normalized values. The model showed better progress in the initial epochs but performed relatively poorly at the end (Fig. 2). Since once the training is finished only the last model will be saved, we used a model monitor to save the best model based on the lowest validation loss. If this model performs well on the test dataset too, we can use it as a working model.

**Fig. 3:**
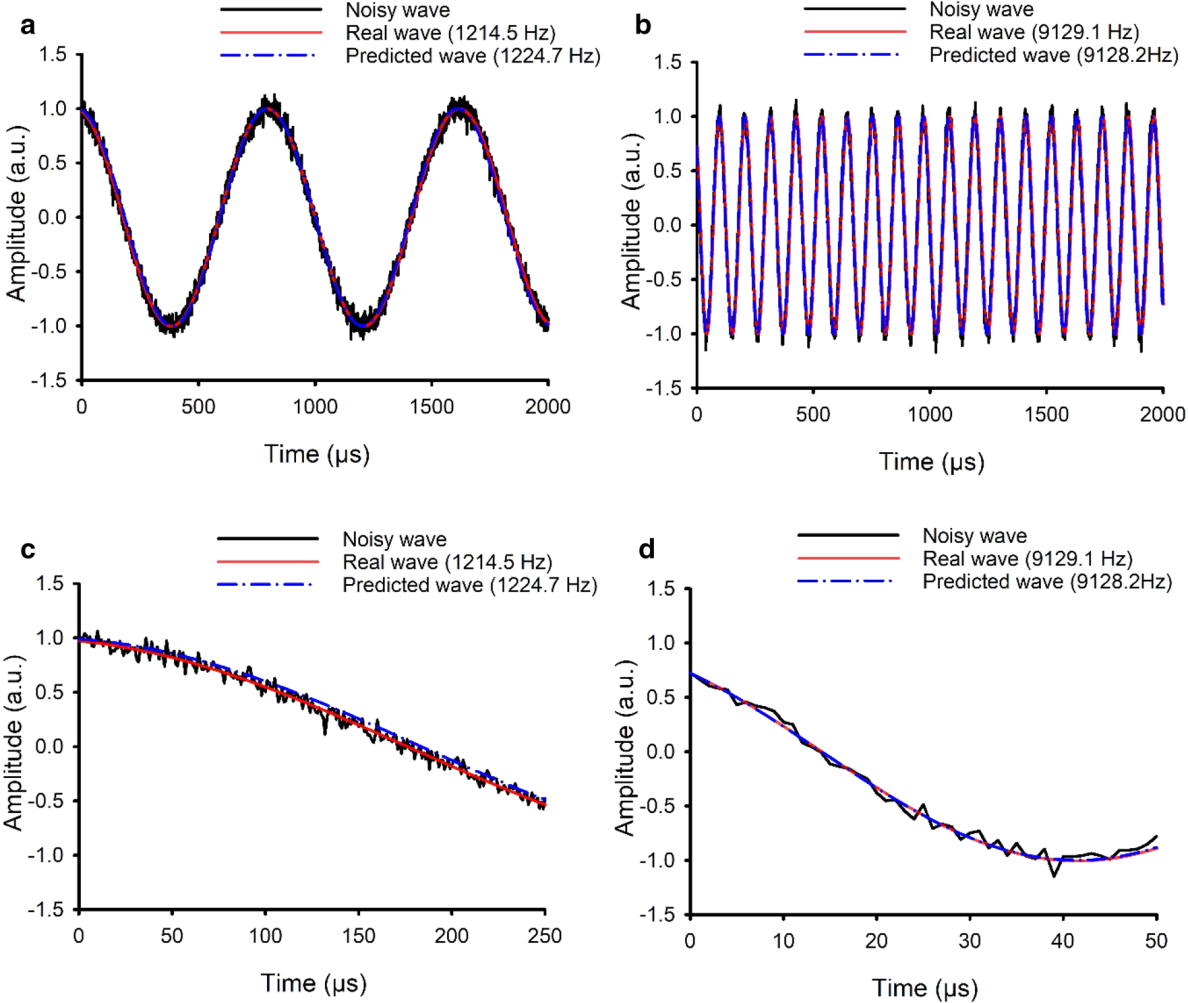
Accuracy of the NN model: examples **a** 1214.5 Hz and **b** 9128.2 Hz, and zoomed views for **c** 1214.5 Hz and **d** 9129.1 Hz

To show the model’s functionality on both low and high frequencies, we present the prediction accuracy of the model at one frequency at the low end of the frequency range and one from the high end. The model performed well on both frequencies. The model predicted 1214.5 Hz at real *f* = 1224.7 Hz (error = 10.2 Hz = 0.83%; Fig. 3a), and 9128.2 Hz at real *f* = 9129.1 Hz (error = 1.1 Hz = 0.012%; Fig. 3b); zoomed views (Fig. 3c, d) show that these error are completely acceptable due to the background noise.

### Generalizing to other frequencies

We can generalize this model for other frequency ranges by a method similar to coordinate transformation. Assume that the time, frequency, and the sine function create a three dimensional space. The idea is to change the time and frequency while keeping the sine function constant. So in (1) if we keep the value *ft* constant, the final results stay valid. We already have the results for 1 kHz to 10 kHz. Let’s assume that we wanted the results for 1 GHz to 10 GHz:

6 $$\begin{aligned} f({\text{KHz}}) \times t({\mu s}) & = f({\text{KHz}}) \times 10^{6} \times 10^{ - 6} \times t({\mu s}) \\ & = f({\text{GHz}}) \times t({\text{ps}}), \\ \end{aligned}$$

**Fig. 4:**
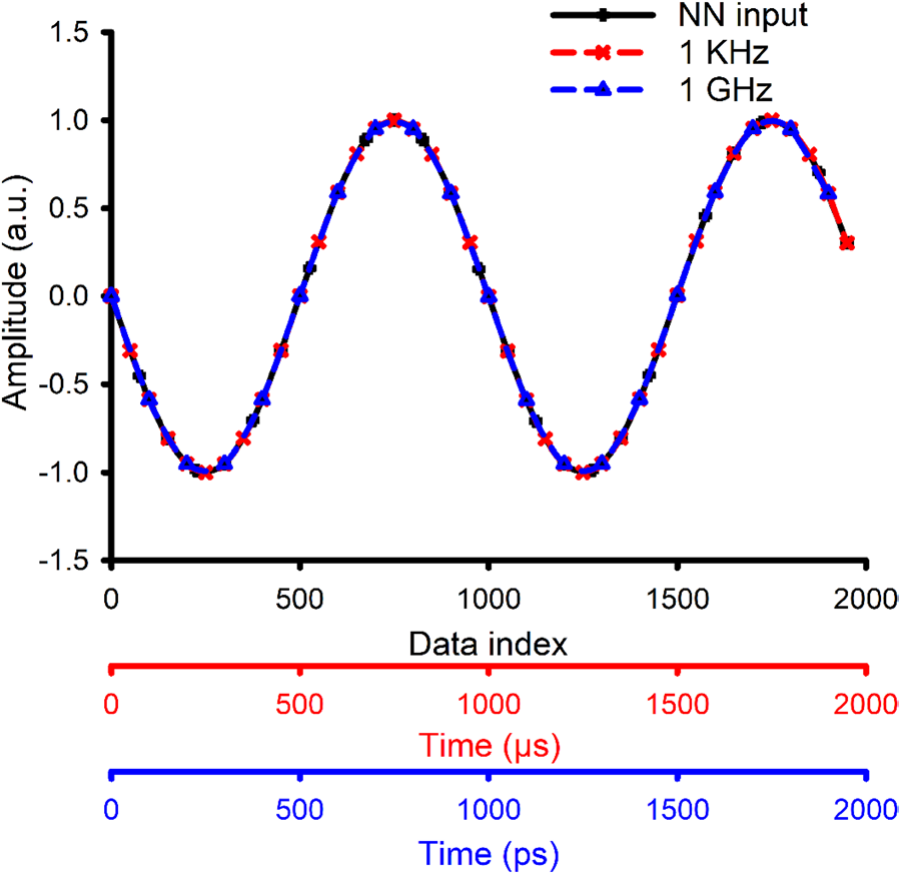
Generalizing the results to other frequencies. A same wave for NN input (black), 1 kHz (red), and 1 GHz (blue). The NN model cannot see the difference between these input waves

So the whole process is transforming the coordinate system to a new one which has different time and frequency axes but the same sine wave. Since the input of the NN model is the sine wave, it cannot distinguish the changes made to the time and frequency, we just need to keep in mind that the new results is for the transformed coordinate system which is in GHz as is shown in Fig. 4.

## Conclusions

We used deep learning to estimate the frequency of a noisy sinusoidal wave. 100,000 noisy waves from 1 to 10 kHz was provided for training, validating and testing the model. We discussed the model architecture and the data preprocessing needed for the model to function efficiently. We investigated the model efficiency on high and low frequencies. The model was able to find the desired frequencies on the unseen data with a very low error, and in a fraction of a second.

## Data Availability

The datasets used and/or analysed during the current study are available from the corresponding author on reasonable request.
